# Enhanced implementation of low back pain guidelines in general practice: study protocol of a cluster randomised controlled trial

**DOI:** 10.1186/1748-5908-8-124

**Published:** 2013-10-20

**Authors:** Allan Riis, Cathrine Elgaard Jensen, Flemming Bro, Helle Terkildsen Maindal, Karin Dam Petersen, Martin Bach Jensen

**Affiliations:** 1 Research Unit for General Practice in the North Denmark region, Aalborg, Denmark; 2 Department of Public Health, Aarhus University, Aarhus, Denmark; 3 Danish Centre for Health Care Improvements, Faculty of Social Sciences and Faculty of Health Sciences, Aalborg University, Aalborg, Denmark; 4 Research Unit for General Practice, Aarhus University, Aarhus, Denmark

**Keywords:** General practice, Intervention studies, Guideline, Health plan implementation, Low back pain, Referral, Consultation

## Abstract

**Background:**

Evidence-based clinical practice guidelines may improve treatment quality, but the uptake of guideline recommendations is often incomplete and slow. Recently new low back pain guidelines are being launched in Denmark. The guidelines are considered to reduce personal and public costs. The aim of this study is to evaluate whether a complex, multifaceted implementation strategy of the low back pain guidelines will reduce secondary care referral and improve patient outcomes compared to the usual simple implementation strategy.

**Methods/design:**

In a two-armed cluster randomised trial, 100 general practices (clusters) and 2,700 patients aged 18 to 65 years from the North Denmark region will be included. Practices are randomly allocated 1:1 to a simple or a complex implementation strategy. Intervention practices will receive a complex implementation strategy, including guideline facilitator visits, stratification tools, and quality reports on low back pain treatment. Primary outcome is referral to secondary care. Secondary outcomes are pain, physical function, health-related quality of life, patient satisfaction with care and treatment outcome, employment status, and sick leave. Primary and secondary outcomes pertain to the patient level. Assessments of outcomes are blinded and follow the intention-to-treat principle. Additionally, a process assessment will evaluate the degree to which the intervention elements will be delivered as planned, as well as measure changes in beliefs and behaviours among general practitioners and patients.

**Discussion:**

This study provides knowledge concerning the process and effect of an intervention to implement low back pain guidelines in general practice, and will provide insight on essential elements to include in future implementation strategies in general practice.

**Trial registration:**

Registered as NCT01699256 on ClinicalTrials.gov.

## Background

The prevalence of low back pain (LBP) in any form is between 25% and 30%. Among LBP patients, about 50% have consulted their general practitioner (GP) for LBP during the last year [1]. LBP generates personal, social, and public costs, and in 2005, Danish costs for treatment, sick leaves, and incapacity benefits were estimated at 16.8 billion DKK (~ 2.3 billion €) [2]. Recently, new clinical practice guidelines were introduced in Denmark, which include advice for primary care on assessment and treatment of LBP patients [3].

The new guidelines are expected to improve overall treatment and they describe the roles of different healthcare providers and define when a GP should refer a patient to a secondary care spine centre. Primary care treatment is considered sufficient for most LBP patients, and secondary referral is usually only recommended if the patient does not improve within eight weeks of primary care treatment. The guidelines include recommendations to the GPs on advising the patients to stay as physically active as possible and to evaluate the need for analgesics and supplementary treatment (*e.g.*, manual therapy or exercises) [3]. More patients are expected to improve by following the guidelines, and referral to secondary care can thus be reduced.

New guidelines are a potential vehicle for changing GP behaviour and securing implementation of evidence-based knowledge into clinical practice. However, implementation strategies with simple, passive diffusion of guidelines, such as simply making newsletters available and other strategies based on information dissemination, have shown insufficient results [4-6]. Consensus regarding the optimal content of components in an intervention strategy has not yet been established, but a review on the effectiveness of clinical guideline implementation suggests the use of a complex approach with active engagement of GPs throughout the process [7]. Activities such as the use of computer-based reminder systems, educational visits, and the use of several activities in combination have shown positive effects in changing GP behaviour [8].

Knowledge concerning how to introduce new guidelines in general practice, however, is sparse, and further knowledge is needed in order to support guideline compliance among GPs and to ensure best evidence-based practice. The aim of this study is to compare a complex implementation strategy with a simple implementation strategy during the implementation of the new LBP guidelines in the North Denmark region. Primary and secondary outcomes pertain to the patient level. A process evaluation pertains to both practice and patient level. A concomitant health economic analysis will be described in a separate protocol.

### Overall theory of changing general practice behaviour

A recently proposed framework for changing clinical behaviour argues that if behaviour change is to be generated, addressing the GP’s capability, opportunity, and motivation is required [9]. The intervention outlined in this study’s protocol supports the GP’s ability to perform an appropriate physical examination and thus enhance GP’s physical capability and thereby support GP’s provision of guidelines. Pop-ups and discussions with guideline facilitators aim to improve the GP’s psychological capability to treat LBP. The practical opportunity for GPs to follow the guidelines is, in our setting, supported through a new referral option for patients with social needs. A social, conducive environment for the process of change is established by conducting practice meetings with guideline facilitators and at small group educational meetings. The GP’s reflective and emotional motivation is addressed through reminders, mouse pads, feedback of monitoring LBP treatment, and discussions with peers and guideline facilitators about the guidelines and the GP’s experiences with the implementation of the guidelines.

Through addressing all of these components, a coordinated set of activities was developed to generate an intervention profile targeted at changing GP behaviour, through better guideline compliance, in order to improve LBP treatment. All activities in this project fall into three categories: capability, opportunity, and motivation (Table 1).

**Table 1:** Activities aimed at changing GP behaviour

**Activities aimed at GPs**	**Capability (physical / psychological)**	**Opportunity (physical / social)**	**Motivation (reflective / automatic)**
**Usual activities (Control and intervention practices)**			
Regional information meetings	X		X
Regional website and written material	X		X
Small group continuing medical education	X		X
**Passive supportive activities (Control and intervention practices)**			
Social medicine referral opportunity		X	
Electronic medical record pop-ups	X		X
Financial incentives			X
Posters reminding of guidelines			X
Mouse pads guiding diagnosis coding, medical record procedures, and reminding of guidelines	X		X
**Pro-active supportive activities (Intervention practices)**			
Facilitator visit	X		X
Feedback/quality assurance	X		X
Info-folder delivered at facilitator visit	X		X
STart Back stratification tool*	X	X	
Social medical screening tool*	X	X	

Activities aimed at changing GP behaviour sorted under capability, opportunity, and motivation. *STarT Back Tool and the SOS screening tool are built into the GPs’ electronic medical record and are filled in at the patient’s first or second consultation regarding LBP.

## Methods/design

### Trial design

**Figure 1:**
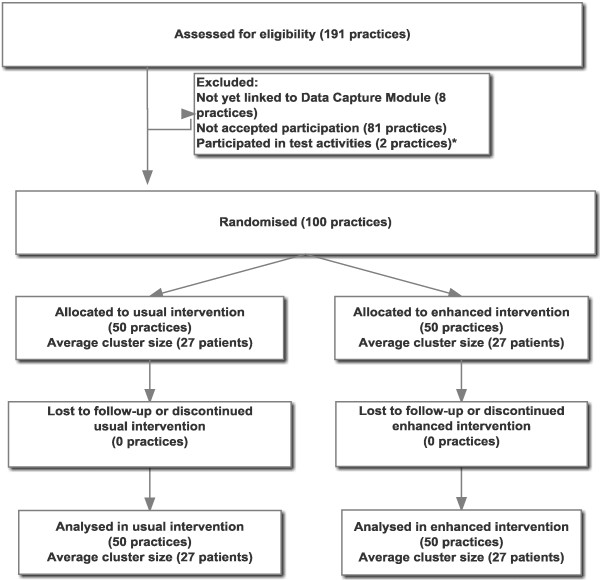
**Chart of expected flow of clusters and patients throughout the trial.** Actual numbers of clusters, average cluster size, and variance of cluster sizes, number of patients contributing data for the primary outcome, as well as number of patients participating with questionnaires will be concluded in reporting of results. ^*^In three practices, single GPs are excluded from participation without excluding the total practice.

This is a two-armed 1:1 cluster randomised controlled trial (CRCT) comparing a control and an intervention group of Danish GP practices. Clusters are defined as patients originating from the same practice. A cluster randomisation was chosen for practical reasons and to prevent possible contamination by asking GPs to treat patients as two groups. The study will include 2,700 patients through stepwise inclusion of 100 practices (Figure 1). In Denmark, almost all secondary-care treatment requires GP referral [10]. All participating GPs receive the usual implementation strategy and passive supportive activities, which include the opportunity to refer patients to an evaluation at the Department of Social Medicine, as well as activities aimed at the inclusion of patients. In addition, intervention practices receive a set of proactive activities including facilitator visits, electronic stratification tools, quality reports, and feedback on LBP treatment.

Study preparation has been aimed at investigating how to change GPs’ behaviours and how to motivate GPs to participate in the study. Single activities such as new stratification and screening tools in the electronic medical record system, electronic pop-ups, facilitator visits, and patient questionnaires have been tested separately prior to this study. In addition, prior to the study, a full-package test involving installation of pop-ups, GP questionnaires, facilitator visit, and facilitator questionnaire was performed, inspired by the stages in 'Interventions to Change the Physician Performance’ [11] (Table 2). Based on small-scale testing in selected practices, we subsequently adjusted the intervention.

**Table 2:** Stages of the intervention

**Stage**	**Purpose**	**Activities**
**Stage 0: preclinical: model development**	To develop an intervention model based on theoretical understanding and empirical research	In the North Denmark region general practice, a complex strategy for new low back pain guideline implementation will be tested to study behaviour change (professional practice) and clinical results (patient outcomes).
**Stage I: testing and remodelling**	Experiments with activities of the intervention in artificial settings	Testing and remodelling structural medical record changes including GP testing of STarT and SOS with patients. Testing and remodelling different questionnaires on volunteers without LBP, GPs, researchers, and patients. Testing and remodelling LBP patient completed questionnaires. Developing electronic generated feedback quality reports for GPs. Testing and remodelling facilitator visits, and feedback procedures on facilitator training sessions.
**Stage II: maximum variation studies**	Full package intervention in selected units of the target group with close monitoring	Full package delivery of facilitator visit at a general practice, use of stratifying tools and changes in the medical record. Inviting patients to electronic or paper version questionnaire. Monitor whether data imputed in medical records and patient questionnaires will be stored at DAK-E, delivered to external database provider, and available for data retrieval.
**Stage III: efficacy studies**	Cluster RCT with ideal intervention, delivery, randomisation, control group and close monitoring	Randomised controlled trial. Inclusion of provider numbers and patients. Use of guideline facilitator visits. Use of stratifying tools. Use of supervising facilitator contacts and feedback quality reports. Monitoring intervention delivery. Monitoring guideline compliance. Monitoring treatment courses. Monitoring GPs skills and beliefs. Measuring secondary care referral and patient related outcomes.
**Stage IV: effectiveness studies**	Routine intervention delivery and ad hoc or routine monitoring	Cost-effectiveness analysis and other health economic analyses are planned and will be described in a separate protocol.

Stages in study development and for intervention performance.

### Eligibility and recruitment

#### ***Inclusion and exclusion criteria for general practices***

General practices in the North Denmark region are eligible for inclusion. Excluded are practices without the electronic data capture program Sentinel, which links the electronic medical record system to the Danish General Practice Database (DAMD), hosted by the Danish Quality Unit for General Practice (by June 2013, 96% were linked to DAMD). Individual GPs will be excluded if they participated in the pilot testing of the study. By October 2011, the North Denmark region had 191 general practice provider numbers representing 332 GPs. The total number of patients in the North Denmark region was 579,829, giving an average of 3,035 listed patients per practice [12].

#### ***Recruitment of general practices***

At local meetings for GPs, one of the investigators (MBJ) informed colleagues about the new guidelines for treatment of LBP and encouraged participation in the study. The GPs will be invited to participate in this study by electronic mail, written letters, and calls from the Quality Unit for General Practice in the North Denmark region (Nord-KAP). To support enrolment, GPs are informed that participation will improve their skills in managing patients with LBP, will help implement the new guidelines, and will provide a new opportunity to refer patients with LBP and concomitant complex social problems to the Department of Social Medicine. In addition, GPs in the control group are paid 1,500 DKK (~200 €) and GPs in the intervention group are paid 2,500 DKK (~333 €) for participation.

#### ***Inclusion and exclusion criteria for patients***

Included are patients aged 18 to 65 years presenting with LBP, with and without leg pain, based on ICPC-2 diagnosis coding L02, L03, L84, or L86 [13]. Excluded are patients with red flags (signs of serious pathology), pregnant women, and patients with insufficient Danish language skills. Excluded patients are registered in DAMD and, together with basic data regarding excluded patients (number, gender, age), will be used to evaluate the selection of patients.

#### ***Recruitment of patients***

For recruiting patients, when a patient with LBP consults a GP, a data-capture program pops up in the electronic medical record. This pop-up contains a question concerning whether the patient agrees to be contacted regarding questionnaire reminders. If the patient agrees, the GP hands out an envelope containing an informational letter about the study, a questionnaire, and a reply envelope with prepaid postage. In the informational letter, patients are encouraged to give informed consent to participate with questionnaires. The consent is given in the first of four questionnaires. Filling in the questionnaire can be done either electronically or by returning the written questionnaire. The general practices will receive posters and mouse pads with LBP diagnoses and pop-up information to remind the GPs of the study. The DAMD database [14] will supply weekly lists of included patients. Reminders will be sent to patients who have agreed to be contacted but who have not filled out a questionnaire. The progress of recruitment will be evaluated every fortnight, and if inclusion is less than one patient per fortnight per participating GP, practices are contacted to discuss reasons for slow inclusion.

#### ***Implementation activities offered to all participating practices***

All practices receive usual guideline implementation, which includes small group continuing medical education meetings, electronic newsletters describing the guidelines [15], and an invitation to participate in regional information meetings. All participating practices are also offered passive supportive activities including restructuring of the electronic medical record, activities reminding of patient inclusion, and the opportunity to refer patients to the Regional Department of Social Medicine for evaluation.

#### ***Implementation activities offered to intervention practices***

In addition to the supportive activities, the intervention practices will receive three additional active supportive activities: guideline for facilitator visit; two patient risk-stratifying tools (Start Back Tool and SOcial risk Screening (SOS) questions; and data feedback on LBP treatment. The following details each of these items:

1. Five primary care physiotherapists are guidelines facilitators. They all have a special certification in LBP assessment and have participated in a ten-hour training course covering the new LBP guidelines in connection with this study. To unify and optimise facilitator visits, the course included lectures and supervised role-playing on how to introduce change in clinical behaviour. The facilitator will visit the practice at inclusion and inform about treatment of LBP according to the new guidelines. Prior to patient inclusion each practice will be offered a one to two hour facilitator visit. After four weeks of patient inclusion, a half-hour follow-up visit or contact by phone or mail will be offered.

2. Two stratifying tools are made available to identify patients at risk of persistent symptoms:

a. The STarT Back Tool divides patients into low, medium, or high risk of prolonged symptoms and guides for treatment [16,17]. In our setting, it is not a requirement that those delivering the treatment for high-risk patients to have specialised training in targeting psychological aspects.

a. The SOS questions (Additional file 1) inquires whether the LBP raises concern about work ability, whether the patient plans to have, or already has, compensation claims or seeks pension, or whether there are any other important issues that could be barriers for recovery. The GPs may use the two risk-stratifying tools at either the initial or the second consultation for LBP. The tools may be viewed in the context of the theory of coloured flags [18] and incorporate biological, psychological, and social aspects. Patients with red flags (serious pathology) are excluded. Yellow flags (beliefs, emotional responses, and pain behaviour) are addressed in the STarT Back Tool. Blue flags (perceptions about the relationship between work and health), black flags (system or contextual obstacles such as legislation, injury claim conflicts, etc.), and orange flags (psychiatric factors) are encompassed in the SOS questions.

3. Feedback on assessment, treatment, and referral of LBP patients during the project: The GPs have access to their own statistics regarding guideline compliance from the DAMD database and may choose to discuss the statistics with their guideline facilitator.

### Data collection

**Figure 2:**
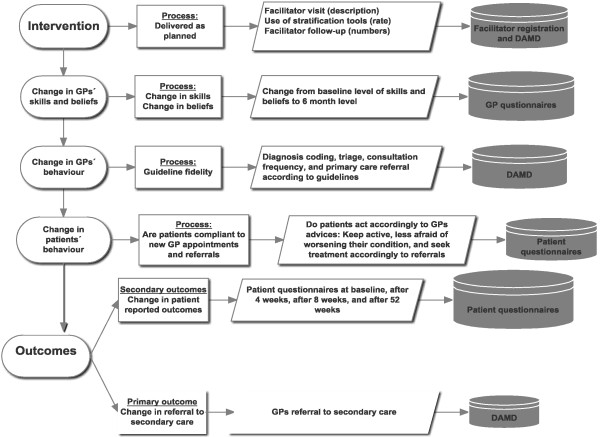
**Illustration of the monitoring procedure.** The first column depicts purpose of measuring; the second column classifies type of measurement; the third column describes type of actions included for measuring; and the fourth column depicts original data storage. All data are merged to the study database.

Collected data for this project will be kept and merged by an external provider using the unique personal identification number (CPR number) that is assigned to all Danish citizens. Data are collected from the guideline facilitators, pop-ups in the GPs electronic medical record (DAMD database), GP questionnaires, patient questionnaires, and regional registries (Figure 2).

### Data capture from the medical record pop-ups

The pop-ups for this project are built into the medical record systems and are activated when the GP enters an ICPC-2 code for LBP [14]. The pop-up at the initial consultation includes questions concerning duration of pain, previous LBP episodes, triage (unspecific LBP, nerve root pain, and red flags/serious pathology), and new appointments. At succeeding consultations, the pop-ups contain questions on symptoms, recommended and applied supplementary treatment, and future planned GP visits. The SOS questions and the STarT Back Tool only appear for the intervention practices and can be filled in either at first or second consultation. Every consultation contains a pop-up question on whether the patient has been referred to secondary care treatment (primary outcome). Pop-ups will cease to appear 12 weeks after the first consultation.

### Patient questionnaires

Patients who wish to participate with questionnaires are requested to fill in a questionnaire immediately after the initial GP consultation and after four, eight, and 52 weeks. In case the patient does not respond to a questionnaire, reminders are sent following one- and two-week delays. In the first questionnaire, patients are asked for baseline characteristics on educational level, co-morbidity, as well as the STarT Back Tool questions. Included in the first questionnaire and repeated in the next three questionnaires are questions on the secondary outcomes and about advice received and advice followed.

### Regional registries, facilitators, and GPs

Data regarding provider numbers and associated GPs will be delivered by the Primary Care Unit in the North Denmark region. The regional Department of Health Planning and Quality provide data on primary care reimbursements (*i.e.*, visits to GPs, physiotherapists, and chiropractors).

Following the initial visit and at later contacts with the intervention clinics, the guideline facilitator will log on to the project database and fill out a questionnaire to record which components of the intervention were delivered (Table 3).

**Table 3:** Registrations from facilitators

**Topics at the initial guideline facilitator visit**	**Only intervention group practices**	
	**Answer**	**Appearance of textbox when answering no.**
Medical history	Yes/No	√
Clinical examination	Yes/No	√
Triage	Yes/No	√
ICPC-coding	Yes/No	√
Patient general advices	Yes/No	√
Re-evaluation	Yes/No	√
STarT Back Tool	Yes/No	√
SOcial Screening questions	Yes/No	√
Supplementary treatment	Yes/No	√
Referral to secondary care	Yes/No	√
Guideline hand-outs	Yes/No	√
Pop-up instructions	Yes/No	-
(If yes) – at the computer screen	Yes/No	-
Duration of visit	Minutes	-
Participants	Numbers	-
Follow-up appointment made	Yes/No	√

Overview of data collection from facilitator visits. The questions are entered into the database by the facilitator after the initial visit. Data concerning dates and topics in following contacts between facilitators and practices will be entered as well.

At inclusion and after six months, GPs are asked to fill out a questionnaire. They are asked about their capability, opportunity, and motivation for LBP treatment and their views on implementation strategies.

### Data completeness, quality, and security

Every fortnight, the study group will perform evaluations of the number of included patients, as well as completion and accuracy of data forms, and act on any problems (missing data, slow inclusion, etc.). Data completeness regarding referral to secondary care treatment (primary outcome) is expected to be between 98 and 100%. Data completeness from patient questionnaires (secondary outcomes) is expected to be about 80%. Paper-version questionnaires will be double keyed into the database. Pop-up data are sent daily to the DAMD database, and from the DAMD to the project database on a weekly basis. The project database is provided with access login and writing recording. Backup copying will be performed daily.

### Primary outcome

The primary outcome measure is GP referral to secondary care within 12 weeks after initial GP appointment. A 5% lower referral rate is considered clinically relevant and is expected in the intervention group compared to the control group. To validate if GPs secondary-care referral results in actual secondary care treatment, data is collected from the North Denmark region administration on diagnoses.

### Secondary outcomes

Changes from baseline to four, eight, and 52 weeks are evaluated for the following secondary outcomes: physical function, evaluated by the 23-questionnaire Roland Morris Patrick (for the individual patient, a three-point improvement is considered clinically relevant); pain score, evaluated by a numerical rating scale from 'no pain’ to 'maximal pain’ (score 0 to 10; a 30% change is considered clinically relevant for an individual patient); health-related quality of life, evaluated by EQ-5D score; patient satisfaction (treatment; a 30% change is considered clinically relevant for an individual patient); patient satisfaction (outcome; a 30% change is considered clinically relevant for an individual patient); employment status (have work, yes/no); and sick leave (number of days).

### Tertiary process outcomes

The following three areas will be described in relation to process evaluation: Did general practice receive the intended implementation activities? Did the intervention change the skills, beliefs, and behaviour of the GPs? Did the intervention change the beliefs and behaviour of the patient? The three areas are elaborated below.

### Evaluating the delivery of the implementation

Actual implementation delivered to interventions practices is monitored by the following items:

1. Delivered intervention at the initial guideline facilitator visit.

2. Use of STarT Back Tool and SOS questions.

3. Follow-up contacts with facilitators.

4. Use of data feedback by the GPs.

### Evaluating skills, beliefs and fidelity of the GP and guideline compliance

1. Skills are evaluated by asking about the GP’s satisfaction with own ability to handle LBP patients and if there are aspects that the GP wants to improve.

2. Beliefs of the GPs are evaluated by six questions on GP agreement with guideline recommendations. GPs are asked questions regarding patient history, patient examination, patient information, advice to stay active, and regarding referral to supplementary treatment in case the patient does not improve.

3. Behaviour of the GPs are evaluated by the referral rate in relation to the risk stratification (STarT Back Tool) and SOS questions, and the proportion of patients who have not improved by four weeks who have been advised to receive supplementary primary care treatment.

### Evaluating the beliefs and behaviour of the patient

Use of supplementary treatment, use of analgesics, and changes in Roland Morris Questions 9 and 23 in patient questionnaires will be used to evaluate changes in patient beliefs and behaviours. Changes will be measured eight weeks after the patient’s initial GP appointment.

### Study preparation

Prior to the study, the complex implementation strategy was developed, tested, remodelled, and retested in order to achieve maximum effect and evaluate the logistical aspects of the study [11].

To evaluate the GPs’ use of LBP diagnoses, a national sample of 624 practices using diagnosis from the past 12 months, including data from the GPs coding >70% of their contacts, was evaluated. The data included 2,000,612 patients and showed the following prevalence of diagnoses: L02, 2.73%; L03, 1.57%; L84, 0.92%; and L86, 0.84% [H. Schroll: Danish Quality Unit for General Practice. December 2012]. With stepwise inclusion of practices, a conservative estimate of duration of patient inclusion over twelve months was made.

To assess additional explanatory variables other than the intervention itself, analyses were performed on other variables. Registry data were obtained on all GPs in the North Denmark region regarding referrals to regional hospitals in 2011. LBP diagnoses (ICD10 diagnoses DM47, DM48, DM51, DM53, and DM54) were included. Analyses of these data showed differences in referral rates for practice size (*t* test of small vs. large practice, *p* = 0.11, Sd test, *p* = 0.023), urbanisation (*t* test of rural vs. mixed, *p* = 0.01), and educational level (Sd test of low vs. high, *p* = 0.02). It was decided to both stratify and adjust for practice size and possibly adjust for level of urbanisation and level of education.

The intervention strategy was developed by the research group based on the COM model [9]. Potential barriers to implementation and possible components that could address these barriers were evaluated in relation to feasibility and cost. The subsequent intervention components were tested in three practices and either rejected or adjusted.

Pop-ups with the STarT Back Tool and the SOS questions were tested in three general practices using different medical record systems. Adjustments were made to ease use and to support GP guideline compliance through the structure of the pop-ups. Mouse pads (A4-format) and posters (A3-format) were tested and produced with help from two GPs at the Quality Unit for General Practice in the North Denmark region (NordKAP). Patient questionnaires were tested both as paper questionnaires and in the electronic form to evaluate different aspects (readability, time use, etc.). In all, 40 patients answered paper questionnaires in different variations. GP questionnaires were tested on three GPs and facilitator registrations were tested after a full-scale facilitator visit test.

Prior to initiating the study, a full-scale evaluation of a facilitator visit was conducted. A practice was represented by four GPs and two nurses. One of the GPs had himself worked as a facilitator in general practice and gave feedback after the test. The two authors AR and MBJ participated in order to make final adjustments for the planned facilitator visits.

The ongoing inclusion of practices over several months was utilised for small adjustments in order to address barriers for patient inclusion and the use of this project’s intervention elements. Inclusion started with four small practices. These practices were followed closely and possible problems were corrected before the inclusion of additional practices.

### Statistics

Analyses will be performed according to the CONSORT guidelines [19]. Data will be analysed according to the intention-to-treat principle. We will obtain descriptive statistics for process measures; baseline characteristics and outcome measures will be presented as mean (*SD*) or numbers (%) with 95% confidence intervals, if normally distributed, or otherwise as medians (quartiles). A Generalised Estimating Equation model with logit link and exchangeable correlation will be carried out. Primary outcome (12-week referral rates) will be analysed in logistic regression models and with respect to provider number cluster effects. Employment status will be analysed by logistic regression models. The other secondary outcomes (Roland Morris Questionnaire, numerical pain rating, EQ-5D, patient satisfaction, and sick leave) will be analysed with practice number as a random effect in a linear mixed effects model with provider number as the intercept in profile analyses.

Primary exposure is allocation group (intervention or control group) stratified by the size of the practices. Following practice number, related baseline variables will be analysed and possibly adjusted for size of practice (≤2,000, 2,001 to 5,000, >5,000 listed patients), urbanity, and degree of received components at baseline (participation in regional information meeting [y/n] and read newsletter [y/n]). The following patient-level-related baseline variables will be analysed and possibly adjusted for educational level, age, gender, co-morbidity, duration of pain, and earlier episodes of LBP.

### Sample size

The control groups’ referral rate to secondary care is expected to be 18%. The intervention groups’ referral rate is expected to be reduced to 13%. Hence, this study is powered to detect a 5% point between groups difference in secondary care referral rates. Considerations of possible cluster effects led us to analyse possible intercorrelations. We analysed whether referral rates were the same between provider numbers in the North Denmark region in 2011. The data did not support any differences in referral rates between provider numbers. Assuming no cluster effect seemed unrealistic, and for that reason, a conservative estimate of a 16% cluster effect was used based on earlier studies on cluster effects in primary care [20]. Study size was estimated on an assumption of 90% power, a 5% level of significance, and a cluster effect of 16%. These assumptions lead to a requirement of 1,321 patients in each group. To allow for size difference between groups, the expected number of included patients was raised to 2,700 patients from 100 practices (Figure 1).

### Randomisation and blinding

Practices are randomised 1:1 to control or intervention group and stratified by list size (≤2,000 patients, 2,001 to 5,000 patients, >5,000 patients) in random permuted blocks of two, four, and six. Randomisation was completed using the Stata program RALLOC. When a general practice signs up for participation, and after giving informed consent, the coordinating secretary assigns a participation number and opens the corresponding sealed envelope with allocation information.

Allocation is not blinded for the GPs, guideline facilitators, or the researchers guiding the facilitators and intervention practices (Research assistant Pia Christine Malmstrøm and MBJ). The researchers collecting and analysing the data (AR and CEJ) are blinded to the randomisation status of the general practice until the statistical analysis has been completed. Patients are told that the general practice is participating in a research project and are invited to participate by filling out patient questionnaires. The patients are told they are participating in a trial. Most patients will not be aware that it is a randomised trial and they will only know of their allocation if the GPs choose to inform them.

### Ethics

The Regional Scientific Ethics Committee and the Danish Health and Medicines Authority did not find that any approval was necessary. The study was registered with the Danish Data Protection Agency, The Danish College of General Practitioners, and at ClinicalTrial.gov (registration number NCT01699256). Patients listed at the included practices are included without patient consent. Written informed consent from the patients for participating with questionnaires will be included in the first questionnaire. Patients may, at any time and without any consequence for their treatment, discontinue participation in the questionnaires. Other than filling out and leaving questionnaires, patients will not suffer any harm or inconvenience. Consent is sought from the participating GPs before the randomisation.

### Trial status

Inclusion of practices started 14 January 2013 and by 2 July 2013; 35 practices and 300 patients are included. Data collection is continuing and is expected to last until the end of Year 2013.

## Discussion

This study will compare two guideline implementation strategies to improve LBP treatment and to increase knowledge about how to implement guidelines in general practice. The study is established in cooperation between the regional bodies involved in planning and implementing the new LBP guidelines and the regional research unit for general practice.

The study is a large cluster randomised controlled trial with expected follow-up regarding the main research question for almost all LBP patients included (referral to secondary care). Patient-related outcome domains are covered in full and extend as recommended in the literature [21]. Development and testing of the intervention was carried out in 2011 and 2012, and a large-scale monitoring process has been developed to optimise outcome measures and also to provide information about the processes between intervention and outcome measuring.

The success of the guideline facilitators in changing GP behaviour in LBP management is uncertain. Physiotherapists have not previously been used as general practice guideline facilitators in the North Denmark region, and how well they will be received is not known. Including GPs and patients in general practice can be a challenging task. The IMPLEMENT study planned to include 92 practices and a total of 2,300 patients. After nine months, however, all 92 practices (112 GPs) but only 29 patients were included, and further recruitment was abandoned [22]. In this study, several actions have been taken to support GP inclusion (financial compensation, information through regional meetings for GPs, letters, and personal contacts) and patient inclusion (posters at GPs, mouse pads for GPs, and automatic diagnosis prompted reminders). Two researchers who are not blinded for randomisation (MBJ and PCM) will be proactive in including more practices and encouraging GPs to hand out envelopes to LBP patients.

This project will study whether the planned intervention is associated with better treatment for LBP patients. The project will also describe which activities should be included in future implementation strategies in primary care for LBP, as well as for other primary care patient groups for whom new guidelines are planned in the near future [15].

## Abbreviations

CRCT: Cluster randomised controlled trial; DAK-E: Danish quality unit of general practice; DAMD: Danish abbreviation for danish general practice database; EQ-5D: European quality of life – 5 dimensions; GP: General practitioner; ICPC: International classification for primary care; LBP: Low back pain; NordKAP: Quality unit for general practice in the North Denmark region; SOS: Social Screening questions; STarT: STarT Back screening tool.

## Competing interests

The authors declare that they have no competing interests.

## Authors’ contributions

MBJ, AR, and FB were responsible for the conception of the study and have together with HTM planned the process analysis. AR, CEJ, KDP, HTM and MBJ formulated and composed the questionnaires. AR, MBJ, and CEJ have performed the daily work with study inclusion and data collection. All authors have been involved in writing the manuscript and all authors have read and approved the final manuscript.

## Supplementary Material

Additional file 1 English translation of the Social medicine screening question. Click here for file

## References

[B1] Macfarlane GJ Beasley M Jones EA Prescott GJ Docking R Keeley P McBeth J Jones GT MUSICIAN Study Team The prevalence and management of low back pain across adulthood: results from a population-based cross-sectional study (the MUSICIAN study) Pain 2012 8 1 27 32 10.1016/j.pain.2011.08.005 21978663

[B2] Koch MB Davidsen M Juel K The Danish national institute of public health, report, May 2011 Danishhttp://www.si-folkesundhed.dk/upload/de_samfundsmæssige_omkostninger_ved_rygsygdom_og_rygsmerter_i_danmark.pdf

[B3] Danish Ministry of Health: Guidelines on LBP treatment Retningslinjer for visitation og henvisning af degenerative lidelser i columna Danish 2010 8 Danish Regions 1 7

[B4] Giguere A Legare F Grimshaw J Turcotte S Fiander M Grudniewicz A Makosso-Kallyth S Wolf FM Farmer AP Gagnon MP Printed educational materials: effects on professional practice and healthcare outcomes Cochrane Database Syst Rev 2012 8 CD004398 23076904 10.1002/14651858.CD004398.pub3 PMC7197046

[B5] Baker R Camosso-Stefinovic J Gillies C Shaw EJ Cheater F Flottorp S Robertson N Tailored interventions to overcome identified barriers to change: effects on professional practice and health care outcomes Cochrane Database Syst Rev 2010 8 3 CD005470 20238340 10.1002/14651858.CD005470.pub2 PMC4164371

[B6] Grimshaw JM Thomas RE MacLennan G Fraser C Ramsay CR Vale L Whitty P Eccles MP Matowe L Shirran L Wensing M Dijkstra R Donaldson C Effectiveness and efficiency of guideline dissemination and implementation strategies Health Technol Assess 2004 8 6 iii iv 1–72 14960256 10.3310/hta8060

[B7] Prior M Guerin M Grimmer-Somers K The effectiveness of clinical guideline implementation strategies–a synthesis of systematic review findings J Eval Clin Pract 2008 8 5 888 897 10.1111/j.1365-2753.2008.01014.x 19018923

[B8] Danish Health and Medicines Authority Map of Medicine pilotprojekt – evalueringsrappport.Danish 2009 12 http://www.sst.dk/Udgivelser/2010/Map%20of%20Medicine%20pilotprojekt%20-%20evalueringsrapport.aspx

[B9] Michie S Van Stralen MM West R The behaviour change wheel: a new method for characterising and designing behaviour change interventions Implement Sci 2011 8 42 10.1186/1748-5908-6-42 21513547 PMC3096582

[B10] Pedersen KM Andersen JS Sondergaard J General practice and primary health care in Denmark J Am Board Fam Med 2012 8 Suppl 1 S34 S38 10.3122/jabfm.2012.02.110216 22403249

[B11] Bro F Rowlands G Interventions to change physician performance: the ChiPP (Change in professional performance) statement Qual Prim Care 2006 8 65 70

[B12] The north Denmark region, praksisudviklingsplan 2012–2015, report Danish 2011 http://www.rn.dk/NR/rdonlyres/395EA932-0B76-4118-8F14-AB9A40E50713/0/UdkastPraksisudviklingsplanforalmenpraksisUdkast.pdf

[B13] Okkes IM Becker HW Bernstein RM Lamberts H The March 2002 update of the electronic version of ICPC-2. A step forward to the use of ICD-10 as a nomenclature and a terminology for ICPC-2 Fam Pract 2002 2002 8 5 543 546 10.1093/fampra/19.5.543 12356710

[B14] Danish Quality Unit of General Practice. About DAK-Ehttp://www.dak-e.dk/flx/en/about-dak-e/

[B15] The north Denmark region: guidelines Danish 2012 http://www.kronikerenheden.dk/NR/rdonlyres/793069EE-2361-40D0-8DA3-71E5685F5521/0/Patientforløbiprimærsektorenforpatientermedlænderygsmerterendeligversionjanuar2012.pdf

[B16] Hill JC Dunn KM Lewis M Mullis R Main CJ Foster NE Hay EM A primary care back pain screening tool: identifying patient subgroups for initial treatment Arthritis Rheum 2008 8 5 632 641 10.1002/art.23563 18438893

[B17] Hill JC Whitehurst DG Lewis M Bryan S Dunn KM Foster NE Konstantinou K Main CJ Mason E Somerville S Sowden G Vohora K Hay EM Comparison of stratified primary care management for low back pain with current best practice (STarT Back): a randomised controlled trial Lancet 2011 8 9802 1560 1571 10.1016/S0140-6736(11)60937-9 21963002 PMC3208163

[B18] Nicholas MK Linton SJ Watson PJ Main CJ 'Decade of the Flags’ working group: early identification and management of psychological risk factors ('yellow flags’) in patients with low back pain: a reappraisal Phys Ther 2011 8 5 737 753 10.2522/ptj.20100224 21451099

[B19] Campbell MK Piaggio G Elbourne DR Altman DG for the CONSORT Group Consort 2010 statement: extension to cluster randomised trials BMJ 2012 8 e5661 10.1136/bmj.e5661 22951546

[B20] Korendijk EJ Hox JJ Moerbeek M Maas CJ Robustness of parameter and standard error estimates against ignoring a contextual effect of a subject-level covariate in cluster-randomized trials Behav Res Methods 2011 8 4 1003 1013 10.3758/s13428-011-0094-8 21512874 PMC3218280

[B21] Bombardier C Outcome assessments in the evaluation of treatment of spinal disorders. Introduction Spine (Phila Pa 1976) 2000 8 24 3097 3099 10.1097/00007632-200012150-00002 11124723

[B22] McKenzie JE French SD O’Connor DA Grimshaw JM Mortimer D Michie S Francis J Spike N Schattner P Kent PM Buchbinder R Green SE IMPLEmenting a clinical practice guideline for acute low back pain evidence-based manageMENT in general practice (IMPLEMENT): cluster randomised controlled trial study protocol Implement Sci 2008 8 11 5908-3-11 10.1186/1748-5908-3-11 18294375 PMC2291069

