# Supplementary material for: Enhanced implementation of low back pain guidelines in general practice: study protocol of a cluster randomised controlled trial
Source: Implement Sci. 2013 Oct 20;8:124. doi: 10.1186/1748-5908-8-124 (PMC4015716; doi:10.1186/1748-5908-8-124)
Supplement: Additional file 1 — English translation of the Social medicine screening question. [file 1748-5908-8-124-S1.pdf]

Additional files

Additional file 1 **Social medicine questions**

EMPLOYMENT STATUS

□ Outside the workforce (Retired, student, etc.)

□ Unemployed

→ Does the LBP raise a concern about return to work?

□ No

□ Yes

If yes, state any action taken

□ Planned contact to a job centre

□ Social medicine referral

□ Occupational medicine referral

□ Other actions (e.g. planned contact to the labour union)

□ No actions taken

□ Employed

→Number of days for sick leave caused by the present episode of LBP

→Does the LBP raise concern about work retention?

□ No

□ Yes

If yes, state any action taken

□ Planned contact to employer or employer representative

□ Planned contact to a job centre

□ Social medicine referral

□ Occupational medicine referral

□ Other actions (e.g. planned contact to the labour union)

□ No actions taken

LEGISLATIVE CLAIMS

□ On-going workers compensation claim

□ No

□ Yes

If yes, state any action taken

□ Advised in general practice

□ Social medicine referral

□ Occupational medicine referral

□ Other actions (e.g. planned contact to the labour union)

□ No actions taken

□ On-going insurance claim

□ No

□ Yes

If yes, state any action taken

□ Advised in general practice

□ Social medicine referral

□ Occupational medicine referral

□ Other actions (e.g. planned contact to the labour union)

□ No actions taken

□ On-going disability pension or other pension claim

□ No

□ Yes

If yes, state any action taken

□ Advised in general practice

□ Social medicine referral

□ Occupational medicine referral

□ Other actions (e.g. planned contact to the labour union)

□ No actions taken

OTHER PERSONAL OR SOCIAL ISSUES INFLUENCING LOW BACK PAIN

□ No

□ Yes

If yes, state which

□ Psychological or psychiatric issues

□ Other health related issues

□ Substance abuse problems

□ Personal or family life problems

□ Other issues (Prompts a textbox)

If yes, state any action taken

□ Advised in general practice

□ Psychologist referral

□ Social medicine referral

□ Other actions (e.g. planned contact to the labour union)

□ No actions taken

Additional file 1**: English translation of the** Social medicine screening question.
